# Antinociceptive and anti-inflammatory effects of a formulation based on *Alpinia zerumbet* essential oil: an *in vivo* and *in vitro* approach

**DOI:** 10.22514/jofph.2025.077

**Published:** 2025-12-12

**Authors:** Wagner Hummig, Julia Maria Zortea, Victor Augusto Benedicto dos Santos, Luiz Eduardo Nunes Ferreira, Juliana Geremias Chichorro

**Affiliations:** ^1^Neurological Institute of Curitiba (INC-PR), 81210-310 Curitiba, PR, Brazil; ^2^Department of Pharmacology, Biological Sciences Sector, Federal University of Parana, 81531-980 Curitiba, PR, Brazil; ^3^Laboratory of Inflammation and Immunology, Guarulhos University, 07011-080 Sao Paulo, SP, Brazil

**Keywords:** Post-operative orofacial pain, Lipopolysaccharide, Heat hyperalgesia, Mechanical hyperalgesia, Inflammation

## Abstract

**Background**: Current pharmacological treatments for acute orofacial pain present limitations, risks, and side effects. Thus, the search for safer alternatives is justified. The essential oil of *Alpinia zerumbet* (EOAz) has been used in the treatment of several medical conditions, including pain and inflammation, but scientific validation is scarce. The aim of this study was to investigate the antinociceptive and anti-inflammatory effects of EOAz in models of acute orofacial pain in rats and in inflammatory parameters *in vitro*. **Methods**: Adult male Wistar rats were subjected to intraoral incision surgery, a model of postoperative pain. The effect of the facial topical application (1 to 3 times a day for 3 days) of EOAz or a formulation based on EOAz (Fb-EOAz) was assessed on heat and mechanical hyperalgesia. The same rats were tested in the open field test (OFT) to assess the influence of the treatments on rats’ locomotion. Moreover, the effects of the treatments were evaluated on facial heat hyperalgesia induced by lipopolysaccharide (LPS), a model of myofascial pain, and *in vitro*, in the release of nitric oxide (NO) and interleukin-6 in macrophages stimulated by LPS. **Results**: EOAz and Fb-EOAz reduced postoperative heat hyperalgesia, but only Fb-EOAz reduced postoperative mechanical hyperalgesia and heat hyperalgesia induced by LPS. Fb-EOAz reduced NO and interleukin-6 release by macrophages stimulated by LPS. None of the treatments affected the rat’s locomotion. **Conclusions**: These data provide preclinical evidence of antinociceptive and anti-inflammatory effects of Fb-EOAz. This approach may represent an alternative or adjuvant therapy in the control of inflammatory and myofascial orofacial pain.

**Figure:** Graphical Abstract

## 1. Introduction

Pain involving the head, face, neck, or intraoral structures is known as orofacial pain, which can have different etiologies and can be acute or chronic [1]. Acute orofacial pain includes pain from both surgical and nonsurgical procedures, and both require effective pain management during treatment and, frequently, pain management also in the postoperative period [2]. Pain after oral surgeries is estimated to affect over 90% of the patients, being considered more intense 5–6 hours after the completion of the procedure and reaching a peak during the first postoperative day [2, 3].

Pharmacological treatment of acute orofacial pain includes the use of non-steroidal anti-inflammatory drugs (NSAIDs, mainly ibuprofen), non-opioid analgesics (paracetamol and dipyrone), and opioid analgesics, which can be prescribed alone or in combination [2, 4]. The prescription of these drugs varies according to the region, with opioids being widely prescribed for acute dental pain in the USA but not in Brazil, where NSAIDs in combination with paracetamol or dipyrone are frequently used [4, 5, 6]. These strategies generally provide satisfactory pain relief but are contraindicated for some patients and associated with risks and side effects. For instance, paracetamol can cause severe hepatotoxicity, dipyrone can induce agranulocytosis, and NSAIDs increase the risk of bleeding and can cause gastrointestinal complications, renal disturbances, and cardiovascular toxicity [7, 8, 9]. Thus, the search for effective and safer therapeutic options that can be used alone or in combination with the currently available therapies is clearly warranted. In this sense, the study of essential oils has gained attention for the treatment of several medical conditions.

*Alpinia zerumbet* (Pers.) B.L.Burtt & R.M.Sm, also known as shell ginger, is a perennial species belonging to the Zingiberaceae family, originating from the East Indies but later introduced in Brazil, where it is commonly found in the northeast region [10, 11]. Multiple parts of *Alpinia zerumbet* have been used for medicinal purposes and for the extraction of essential oils. In Brazil, the medicinal use of *Alpinia zerumbet* by the population includes the treatment of rheumatism, viral infections, cardiovascular disease, high blood pressure, gastric lesions, pain, and inflammation [10]. Likewise, there is an extensive list of biological and pharmacological activities of *Alpinia zerumbet* essential oil that vary according to the part of the plant used (*i.e.*, leaves, rhizomes, fruits, seeds, and flowers), probably due to differences among the chemical components and/or the accumulation of active compounds in each part [11, 12]. However, many of these indications lack scientific validation, and thus pre-clinical and clinical studies conducted with quality and rigor are needed.

The present study aimed to investigate the antinociceptive effect and anti-inflammatory mechanism of a commercial formulation based on *Alpinia zerumbet* essential oil in models of acute orofacial pain in rats and in *in vitro* inflammation assays. The main reasons to test a commercial formulation are its availability for health care providers and the general population, and the quality control in the manufacture of the essential oil, which ensures its safety, purity, and efficacy. Moreover, according to the provider of the commercial formulation, the volume of prescriptions for this product is increasing among dentists for the purpose of treating painful orofacial conditions, but this indication is not on the label and lacks more detailed scientific evidence.

## 2. Material and methods

### 2.1 Animals

Experiments were conducted on 218 adult male Wistar rats (*Rattus norvegicus*) weighing 200 to 250 g. The number of animals per group was determined through a pilot study and the G*Power software (v. 3.1.9.7, Heinrich-Heine-Universität Düsseldorf, Düsseldorf, NRW, Germany). Rats were housed four per cage in a climate-controlled room at 22 ± 2 °C on a 12-hour light/dark cycle with food and water *ad libitum* and wood shavings changed on alternate days. The animals were provided by the vivarium of the Federal University of Parana, and all protocols were previously approved by the Research Ethics Committee for the Use of Animals in the Biological Sciences Sector of the Federal University of Parana (CEUA/BIO-UFPR #1588), all in accordance with the Brazilian guideline of the National Council for the Control of Animal Experimentation (CONCEA) and the Animal Research: Reporting of *In Vivo* Experiments (ARRIVE) guidelines.

### 2.2 Drugs and reagents

The study used: Essential oil of *Alpinia zerumbet* (EOAz, obtained by steam distillation from the stem and leaves in Pernambuco, state of northeastern Brazil, before the flowering period; Hebron Farmacêutica, Brazil), a formulation based on the Essential oil of *Alpinia zerumbet* (Fb-EOAz, commercial name Ziclague, containing 0.08 mL/mL essential oil of *Alpinia zerumbet*, Hebron Farmacêutica, Caruaru, PE , Brazil, ANVISA registration #1155700690025), Vehicle (Fb-Vehicle, related to the formulation of Ziclague; Hebron Farmacêutica, Brazil), Avocado oil (inert oil, Quinarí, Ponta Grossa, PR, Brazil), Ketamine Hydrochloride (047/23, Syntec Tecnologia Farmacêutica Aplicada à Medicina Veterinária, Santana de Parnaíba, SP, Brazil), Xylazine Hydrochloride (034/22, Syntec Tecnologia Farmacêutica Aplicada à Medicina Veterinária, Santana de Parnaíba, SP, Brazil), Isoflurane (006/23, Syntec Tecnologia Farmacêutica Aplicada à Medicina Veterinária, Santana de Parnaíba, SP, Brazil), Sterile saline solution 0.9% (2132191, Equiplex Indústria Farmacêutica, Aparecida de Goiânia, GO, Brazil), Lipopolysaccharide from *Pseudomonas aeruginosa* (LPS) (L9143, Sigma-Aldrich, St. Louis, MO, USA), Enzyme-Linked Immunosorbent Assay (ELISA) kit for interleukin (IL) dosage (330449, DuoSet, Mouse IL-6, R&D System, Minneapolis, MN, USA), Thiazolyl Blue Tetrazolium Bromide (MTT) reduction assay (MKCR1832, Sigma-Aldrich, St. Louis, MO, USA), and Griess Reagent (0000646727, Griess Reagent System, Promega Corporation, Madison, WI, USA).

### 2.3 Orofacial postoperative pain model

The orofacial postoperative pain model was previously described by our group [13, 14, 15]. Initially, the animal was anesthetized with an intraperitoneal (i.p.) injection of ketamine and xylazine (50 mg/kg and 10 mg/kg, respectively). For the incision of the buccal mucosa, the animal was positioned laterally for the insertion of an intraoral device that kept its mouth open throughout the procedure. Using a scalpel blade #5, an intraoral incision (2 mm × 10 mm) was made on the right side of the animals’ mucosa. After the incision, the mucosa was sutured with a 5-0 silk thread with just a single stitch in the middle of the incision. The sham animals were subjected to the same procedures, but the incision and suture were not performed. After the surgery, the animals were monitored in a heated room until full anesthesia recovery.

### 2.4 LPS-induced myofascial pain

Animals were anesthetized via inhalation with halothane (1.5 L/minute), and a small area of the facial region was trichotomized, preserving the vibrissae. Then, the animals received an injection of LPS (10 μg/50 μL) or vehicle (0.9% sodium chloride, 50 μL) directly into the masseter muscle. The injections were repeated on two consecutive days. This method of anesthesia allows rapid recovery of the animals so that behavioral evaluations can be made. This protocol was based on previous studies with some modifications [14, 16].

### 2.5 Orofacial heat hyperalgesia assessment

Facial heat hyperalgesia was evaluated in rats as previously reported by our group [13, 14, 15]. First, rats were habituated to the restraining method to avoid stress during the test. Facial heat sensory threshold was assessed by the approximation of a radiant heat source (about 50 °C) 1 cm from the surface of the right vibrissa whisker pad area. The response latency to display either head withdrawal or vigorous flicking of the snout was recorded. A cut-off time of 20 seconds was established to prevent tissue damage.

### 2.6 Orofacial mechanical hyperalgesia assessment

This assessment was performed as previously described by our group [14, 15]. First, rats were maintained individually in acrylic boxes (30 cm^3^) for at least 2 hours for habituation. The baseline mechanical threshold was measured using a graded series of 8 von Frey filaments ranging from 0.04 to 8.0 g in increasing order (Semmes-Weinstein monofilaments, Stoelting, Wood Dale, IL, USA). Each filament was applied near the center of the right vibrissa pad, to the point of bending, three times on the same side until it evoked two positive responses, including brisk head withdrawal, facial grooming, or sharp escape or attack reactions against the filament. Only rats that did not react to the application of the 8 g filament in the baseline measure were included in this study to avoid unspecific responses.

### 2.7 Open field

This test was performed as previously described [15, 17, 18] and consisted of placing the rats in the center of an arena (50 cm long, 50 cm wide, and 40 cm high) with closed sidewalls and the floor divided into nine quadrants. Rats’ behavior was recorded for 5 minutes for posterior behavior evaluation using the software Any-maze 7.48 (Stoelting Co., Wood Dale, IL, USA) for Windows, from which the total distance traveled, average speed, time spent by the animals in the center zone of the field, track plot, and average heat plot from the groups for the entire duration of the tests were extracted.

### 2.8 Experimental design

#### 2.8.1 Experiment 1: effect of EOAz and Fb-EOAz in the orofacial postoperative pain model

First, the baseline response to heat and mechanical stimulation was assessed. Then rats were subjected to trichotomy in the facial region to be stimulated, followed by a sham or incision procedure. The trichotomy was performed in the masseter muscle region, on the same side of the incision, to facilitate the treatments’ application and to ensure penetration. On days 1, 2 and 3 after the surgical procedure, the rats received topical treatment (60 μL, applied with a micropipette) of EOAz, Fb-EOAz, Fb-Vehicle, or avocado oil. For the evaluation of heat hyperalgesia, the animals were treated once a day (8 AM), twice a day (8 AM and 12 PM), or three times a day (8 AM, 12 PM and 4 PM), and for the evaluation of mechanical hyperalgesia, they were only treated three times a day (8 AM, 12 PM and 4 PM). The heat and mechanical thresholds of the animals were assessed daily, 30 minutes after the last application, until the fourth day after the surgery.

#### 2.8.2 Experiment 2: effect of EOAz and Fb-EOAz in the open field test (OFT)

On day 3 after the surgical procedure, after the third application of the different compounds (EOAz, Fb-EOAz, Fb-Vehicle, or avocado oil), the animals were transferred to the open field arena. The animals’ behavior was recorded for 5 minutes for posterior analysis.

#### 2.8.3 Experiment 3: effect of EOAz and Fb-EOAz in LPS-induced myofascial pain

After LPS or vehicle (0.9% sodium chloride) injection into the masseter muscle, the animals received topical treatment (60 μL applied in the trichotomized facial region with a micropipette) of EOAz, Fb-EOAz, Fb-Vehicle, or avocado oil 3 times a day (8 AM, 12 PM and 4 PM) for 3 days. The heat threshold of the animals was evaluated daily, before the application of LPS or vehicle on days 1 and 2, and 30 minutes after the treatments on days 1, 2 and 3.

#### 2.8.4 Experiment 4: *in vitro* effects of EOAz and Fb-EOAz in macrophages stimulated by LPS

The protocols were based on previous publications [19, 20]. Immortalized mouse macrophages (RAW 264.7) were cultured in Roswell Park Memorial Institute (RPMI) medium supplemented with 10% Fetal Bovine Serum (FBS), sodium bicarbonate (1500 mg/L), penicillin (100 U/mL), streptomycin (100 μg/mL), and gentamicin (10 μg/mL). Cells were maintained under humidified atmosphere with 5% CO_2_ at 37 °C. The release of NO was determined in the supernatants of cell culture. Cells were seeded in 96-well cell culture plates (1 × 10^5^ cells/well) and incubated for 24 h. Then, the cells were treated with Fb-EOAz (4 and 40 μg/mL) or Fb-Vehicle and incubated for 1 h (37 °C and 5% CO_2_). The inflammatory response was stimulated with 0.5 μg/well of *E. coli* serotype LPS (O111:B4). The plates were then incubated for 24 h at 37 °C and 5% CO_2_. The positive control was performed with cells treated only with LPS, and cells not stimulated were used as a negative control. NO production was determined by the presence of nitrites in the cell culture supernatant using Griess reagent, following the manufacturer’s instructions (0000646727, Griess Reagent System, Promega Corporation, Madison, WI, USA). Results were expressed in nitrite concentration (μM). The cell viability was evaluated by the MTT reduction assay. Briefly, the wells were washed with phosphate buffered saline buffer (PBS; pH 7.4) and 200 μL of RPMI with MTT sodium salt (0.5 mg/mL) were added. The plates were incubated for 4 h (37 °C and 5% CO_2_) for MTT reduction. Optical density was measured using a microplate reader set at 570 nm wavelength. The results were expressed in a percentage of viable cells. For the quantification of interleukin 6 (IL-6) levels, RAW 264.7 cells were seeded in 24-well cell culture plates (3 × 10^5^ cells/well) and incubated for 24 h (37 °C and 5% CO_2_). Cells were treated with Fb-EOAz (4 and 40 μg/mL) or Fb-Vehicle and after 1 h, 2.5 μg/mL LPS from *E. coli* serotype (O111:B4) was added, and the cells were incubated for an additional 24 h. Supernatants were collected, and the level of IL-6 was measured. The quantification of IL-6 was performed using an ELISA kit, according to the protocols provided by the manufacturer (DuoSet, Mouse IL-6, R&D System, Minneapolis, MN, USA). The result was expressed in pg/mL.

### 2.9 Statistical analysis

Data normality was tested using the Shapiro-Wilk test. Repeated-measures two-way Analysis of Variance (Two-way ANOVA) test with Bonferroni *post hoc* were used for the assessment of orofacial heat and mechanical hyperalgesia, and Kruskal-Wallis tests with Dunn-Bonferroni *post hoc* were used for the assessment of the open field. Statistical analyses were performed using JASP for Windows software (JASP Team, Version 0.19.1), graphs were made using GraphPad Prism 10.3.0 for Windows (GraphPad Software, Boston, MA, USA) and *p* values < 0.05 were considered statistically significant.

## 3. Results

### 3.1 Influence of EOAz and Fb-EOAz in heat and mechanical hyperalgesia in a model of orofacial postoperative pain

The development of orofacial heat hyperalgesia after intraoral incision was evaluated with treatments administered topically once, twice, and three times each day. There was no significant difference between sham-operated groups that received the different treatments (*F*(3, 20) = 0.803, *p* = 0.507, ω^2^ = 0.000; Fig. 1A). The incision caused significant orofacial heat hyperalgesia, since there was a significant difference on days 2, 3, and 4 when comparing the Sham + Fb-Vehicle group with the Incision + Fb-Vehicle group (day 2: Mean Difference (MD) = −2.750, Standard Error (SE) = 0.470, *p* < 0.001; day 3: MD = −3.000, SE = 0.386, *p* < 0.001; day 4: MD = −1.750, SE = 0.413, *p* = 0.002). However, a single daily treatment with EOAz and Fb-EOAz did not show an anti-hyperalgesic effect (*F*(3, 20) = 18.253, *p* < 0.001, ω^2^ = 0.381; Fig. 1B).

Fig. 1C illustrates the development of orofacial heat hyperalgesia after the incision (*F*(3, 20) = 5.578, *p* = 0.006, ω^2^ = 0.141; Fig. 1C) when comparing the Sham + Fb-Vehicle group with the Incision + Fb-Vehicle group (day 2: MD = −1.583, SE = 0.493, *p* = 0.026; day 3: MD = −2.417, SE = 0.620, *p* = 0.005; day 4: MD = −1.750, SE = 0.378, *p* < 0.001). EOAz treatment performed twice a day, on days 2 and 3, caused a significant reduction of orofacial heat hyperalgesia when compared to the Incision + Fb-Vehicle group (MD = −1.500, SE = 0.378, *p* = 0.005).

Likewise, the development of orofacial heat hyperalgesia is depicted on Fig. 1D (*F*(3, 20) = 8.707, *p* < 0.001, ω^2^ = 0.216; Fig. 1D), when comparing the Sham + Fb-Vehicle group with the Incision + Fb-Vehicle group (day 2: MD = 2.417, SE = 0.452, *p* < 0.001; day 3: MD = 2.833, SE = 0.456, *p* < 0.001; day 4: MD = 1.417, SE = 0.452, *p* = 0.031). Both treatments, EOAz and FB-EOAz, applied topically 3 times a day, caused significant reduction of orofacial heat hyperalgesia. On day 2, there was a statistical difference when comparing the Incision + Fb-Vehicle group with the Incision + EOAz group (MD = −1.500, SE = 0.452, *p* = 0.021), and on day 3, when comparing Incision + Fb-Vehicle with Incision + Fb-EOAz (MD = −3.167, SE = 0.456, *p* < 0.001) and Incision + EOAz (MD = −1.500, SE = 0.456, *p* = 0.022).

**Fig. 1.** Effect of EOAz and Fb-EOAz on orofacial heat hyperalgesia in a postoperative pain model. The latency for response to heat was assessed in sham and incision rats before (BL) and once a day after the treatments. (A) Sham rats received avocado oil, Fb-Vehicle, EOAz, or Fb-EOAz 3×/day for 3 days. (B) Incision rats received Fb-Vehicle, EOAz, or Fb-EOAz 1×/day. (C) Incision rats received Fb-Vehicle, EOAz, or Fb-EOAz 2×/day. (D) Incision rats received Fb-Vehicle, EOAz, or Fb-EOAz 3×/day. All treatments were repeated for 3 days, and after the last treatment, the heat hyperalgesia was assessed daily. Data are expressed as mean with SEM (n = 6). *p* < 0.05 when compared with: *Sham + Fb-Vehicle; ^#^Incision + Fb-EOAz, and ^&^Incision + EOAz groups. Two-way ANOVA followed by the Bonferroni *post hoc* test. EOAz: essential oil of *Alpinia zerumbet*; Fb-EOAz: formulation based on the EOAz; SEM: standard error of the mean.

The assessment of orofacial mechanical hyperalgesia was performed with treatments administered three times each day for 3 consecutive days. Fig. 2A illustrates that there was no significant difference between sham-operated groups that received the different treatments (*F*(3, 28) = 1.087, *p* = 0.371, ω^2^ = 0.002). The comparison between Sham + Fb-Vehicle and Incision + Fb-Vehicle groups showed that intraoral incision (Fig. 2B) induced a significant reduction of the mechanical threshold on day 2 (MD = 5.880, SE = 1.274, *p* < 0.001), on day 3 (MD = 6.775, SE = 1.127, *p* < 0.001), and on day 4 (MD = 5.025, SE = 1.405, *p* = 0.008). On day 3, Fb-EOAz caused a significant reduction of mechanical hyperalgesia when compared to Incision + Fb-Vehicle (MD = −4.150, SE = 1.127, *p = *0.006).

**Fig. 2.** Effect of EOAz and Fb-EOAz on orofacial mechanical hyperalgesia in a postoperative pain model. The orofacial mechanical hyperalgesia was assessed in sham and incision rats before (BL) and once a day after the treatments. (A) Sham rats received avocado oil, Fb-Vehicle, EOAz, or Fb-EOAz 3×/day for 3 days. (B) Incision rats received Fb-Vehicle, EOAz, or Fb-EOAz 3×/day for 3 days, and after the last treatment, the mechanical hyperalgesia was assessed. Data are expressed as mean with SEM (n = 8). *p* < 0.05 when compared with: *Sham + Fb-Vehicle; ^#^Incision + Fb-EOAz. Two-way ANOVA followed by the Bonferroni *post hoc* test. EOAz: essential oil of *Alpinia zerumbet*; Fb-EOAz: formulation based on the EOAz; SEM: standard error of the mean.

### 3.2 Effect of EOAz and Fb-EOAz in LPS-induced myofascial pain and inflammatory parameters *in vitro*

Fig. 3A illustrates that LPS induced orofacial heat hyperalgesia on day 3 (Vehicle + Fb-Vehicle compared to LPS + Fb-Vehicle: MD = −1.700, SE = 0.437, *p* = 0.006). Repeated treatment with Fb-EOA caused a significant reduction of LPS-induced orofacial heat hyperalgesia on day 3 (MD = −1.450, SE = 0.437, *p* = 0.022).

For the evaluation of the *in vitro* effect in cell culture using RAW 264.7 macrophages, the formazan reduction test was necessary to investigate whether the treatment concentrations could interfere with cell viability and, consequently, in the subsequent evaluation of nitric oxide dosage. In cell viability, the EOAz group was the only one that showed a statistically significant difference compared to the control (*p* < 0.0001, Fig. 3B), indicating cytotoxicity. Therefore, the influence of EOAz on the levels of NO and IL-6 was not investigated, since the interpretation of the results would be impaired by its cytotoxic effect. Fb-Vehicle and Fb-EOAz (4 and 40 μg/mL) did not change the cell viability compared to the control group (Fig. 3B), but both treatments significantly reduced NO release compared to the LPS group (Fb-Vehicle: *p* < 0.0001; Fb-EOAz 4 μg/mL: *p* = 0.0006; Fb-EOAz 40 μg/mL: *p* < 0.0001; Fig. 3C). Likewise, both treatments significantly reduced IL-6 release by macrophages stimulated with LPS, but Fb-EOAz caused a concentration-dependent effect (Fb-Vehicle: *p* < 0.0001; Fb-EOAz 4 μg/mL: *p* = 0.0098; Fb-EOAz 40 μg/mL: *p* < 0.0001; Fig. 3D).

**Fig. 3.** Effect of EOAz and Fb-EOAz in LPS-induced myofascial pain and inflammatory parameters *in vitro*. The latency for response to heat was assessed before (BL) and once a day after the treatments. (A) Rats received LPS or vehicle injection into the masseter muscle followed by the topical treatment with Fb-Vehicle, EOAz, or Fb-EOAz 3×/day for 3 days. (B) Cell viability of RAW 264.7 cells exposed to LPS alone or combined with Fb-Vehicle, EOAz, Fb-EOAz (4 μg/mL), or Fb-EOAz (40 μg/mL). (C) Release of NO by RAW 264.7 cells stimulated with LPS and exposed to Fb-Vehicle, Fb-EOAz (4 μg/mL), or Fb-EOAz (40 μg/mL). (D) Release of IL-6 in RAW 264.7 macrophages stimulated with LPS and treated with Fb-Vehicle, Fb-EOAz (4 μg/mL), or Fb-EOAz (40 μg/mL). Data are expressed as: (A) mean with SEM (n = 5–6); (B–D) mean with SD (n = 5–7). *p* < 0.05 when compared with: (A) *Vehicle + Fb-Vehicle; ^#^LPS + Fb-EOAz. Two-way ANOVA followed by the Bonferroni *post hoc* test; (B–D) *Control; ^&^LPS. ANOVA followed by the Tukey *post hoc* test. EOAz: essential oil of *Alpinia zerumbet*; Fb-EOAz: formulation based on the EOAz; LPS: lipopolysaccharide; NO: nitric oxide; IL-6: interleukin 6; SEM: standard error of the mean.

### 3.3 Open field test

Fig. 4A is a representative track plot report of a single sham rat recorded during the 5-minutes test sessions. For the sham group, there was no statistical difference in the evaluation of the distance traveled (H(3) = 1.887; *p* = 0.596; rank ƞ^2^ = 0.000; Fig. 4B) and in the average speed (H(3) = 1.718; *p* = 0.633; rank ƞ^2^ = 0.000; Fig. 4C) of sham rats that received avocado oil, Fb-Vehicle, EOAz, or Fb-EOAz. However, there was a significant difference in the time in the center zone (H(3) = 11.347; *p* = 0.010; rank ƞ^2^ = 0.417; Fig. 4D) between the Sham + EOAz and Sham + Avocado oil groups (*z* = −3.266; *p* = 0.001, *p_bonf_* = 0.0065).

Fig. 4E is a representative track plot report of a single incision rat recorded during the 5-minutes test sessions. For the incision group, there was no difference between the Sham + Fb-Vehicle and Incision + Fb-Vehicle groups, considering all parameters, and there was no influence of any treatment on the distance traveled (H(3) = 7.620; *p* = 0.055; rank ƞ^2^ = 0.231; Fig. 4F), average speed (H(3) = 7.411; *p* = 0.060; rank ƞ^2^ = 0.221; Fig. 4G), and time in the center zone (H(3) = 2.576; *p* = 0.462; rank ƞ^2^ = 0.000; Fig. 4H).

**Fig. 4.** Effect of EOAz and Fb-EOAz in the open field test. On day 3 after the surgical procedure and after the third application of the different compounds, the rats were tested in the open field. Sham rats received avocado oil, Fb-Vehicle, EOAz, or Fb-EOAz. Panels A and E are representative track plot reports of a single rat recorded during the 5-minutes test sessions, sham and incision rats, respectively, in which it was assessed: (B,F) total distance traveled, (C,G) average speed and (D,H) time in the center zone. Rats subjected to intraoral incision received Fb-Vehicle, EOAz or FB-EOAz. Data are expressed as the minimum and maximum value (whiskers), 1st and 3rd quartile, and the median (n = 6). Statistical difference when *p* < 0.05. Kruskal-Wallis followed by the Dunn-Bonferroni *post hoc* test. EOAz: essential oil of *Alpinia zerumbet*; Fb-EOAz: formulation based on EOAz.

## 4. Discussion

The results of the present study showed that the EOAz and a formulation based on EOAz present anti-hyperalgesic effects in a model of post-operative orofacial pain. The effect seems to be cumulative, since at least 2 topical applications are necessary for efficacy. However, only the commercial formulation significantly reduced the muscle hyperalgesia induced by LPS. This effect can be related to an anti-inflammatory activity of the compound, since it reduced *in vitro* NO release and interleukin-6 in macrophages stimulated with LPS. Finally, it was shown that both formulations did not change the locomotory behavior of the animals.

Around the globe, over 300 million surgeries are performed annually, with pain being the most prevalent postoperative symptom. Despite the advances in medical research, postoperative acute pain management has several limitations and potential side effects, and the preclinical search for novel alternatives for pain control is clearly justified [21]. The orofacial postoperative pain model has been characterized by our group and others [13, 14, 15, 22]. It has been shown that after intraoral incision, rats develop orofacial heat and mechanical hyperalgesia, which peak on day 3 after the surgery and resolve within 5 days. Both heat and mechanical hyperalgesia are susceptible to control by systemic opioids (morphine and codeine), non-opioid analgesics (acetaminophen), and NSAIDs (ibuprofen) [14, 15], demonstrating correspondence with the clinical setting. Herein, we have shown that the topical application of EOAz twice a day caused a significant reduction of orofacial heat hyperalgesia on day 4, while topical application 3 times a day resulted in an anti-hyperalgesic effect on days 2 and 3. For the commercial formulation (Fb-EOAz), it was necessary to have 3 daily applications to obtain a significant reduction of heat hyperalgesia on day 3. However, only Fb-EOAz reduced orofacial mechanical hyperalgesia on postoperative day 3 when applied topically 3 times a day. These results suggest an advantage of the commercial formulation compared to the essential oil. Heat and mechanical hyperalgesia are frequently associated with postoperative pain, but it is well established that they are driven by distinct mechanisms [14, 15, 21, 22, 23]. Thus, the effect of the commercial formulation on heat and mechanical hyperalgesia indicates that it can target a common signaling pathway for both sensory alterations or that it has multiple mechanisms or targets. Consistent with our observations, previous studies have demonstrated that the essential oil from the leaves of *Alpinia zerumbet* administered systemically (30 to 300 mg/kg) caused significant antinociceptive effects in models of nociceptive and acute inflammatory pain [24]. The authors suggested that the antinociceptive effect is partially mediated by opioid receptors, since it was reduced by naloxone, but they also suggested an anti-inflammatory mechanism that needs further investigation. In line with this idea, the systemic administration of an extract from the leaves of *Alpinia zerumbet* (200 and 400 mg/kg) reduced carrageenan-induced edema and leukocyte migration, and acetic acid-induced writhing and vascular permeability in mice [25]. The authors showed, by *in vitro* assays, that some of the mechanisms that contribute to these effects are the antioxidant activity of the extract and a potent inhibition of cyclooxygenase 1 and 2 (COX-1 and COX-2) enzymes [25]. There are many other mechanisms proposed to underlie the anti-inflammatory effects of *Alpinia zerumbet*, but it is worth mentioning that the biological and pharmacological activities of the extracts and essential oils prepared from different parts of the plant present great variation, probably due to differences among the chemical components and/or the accumulation of active compounds in the individual parts [11]. Thus, we focused on comparing our data only with studies that also used the leaves of *Alpinia zerumbet* in the preparation of the extract or essential oil. Herein, a potential anti-inflammatory effect of EOAz and of Fb-EOAz was investigated *in vivo* and *in vitro* in the inflammatory response induced by LPS, a component of the cell wall of gram-negative bacteria. LPS is considered an exogenous ligand of Toll-like Receptor 4 (TLR4), whose activation leads to the stimulation of nuclear factor-κB (NF-κB). This pathway is responsible for regulating the transcription of many pro-inflammatory cytokines that contribute to the development of inflammatory hyperalgesia, as well as stimulating the release of several direct-acting hyperalgesic mediators [14, 26, 27]. Moreover, TLR4/NF-κB signaling is not only activated in response to LPS but also contributes to the development of heat and mechanical hyperalgesia in models of postoperative pain [14, 26, 27]. According to our results, Fb-EOAz, but not EOAz, caused a significant reduction of LPS-induced hyperalgesia after injection into the masseter muscle. This approach was used because the Fb-EOAz is indicated on the label as an adjuvant treatment for muscle spasticity, but muscle pain relief has also been reported with repeated treatment. In the clinical setting, Fb-EOAz is also used topically, being recommended to be sprayed 1 to 6 times a day on the affected muscle. Taken together, the effects of Fb-EOAz in LPS-induced hyperalgesia and in the postoperative pain model indicate anti-inflammatory properties of the formulation. Thus, its influence on the release of NO in macrophages stimulated with LPS was investigated, since NO has been used as a marker to estimate the anti-inflammatory activity of different compounds [28]. Fb-EOAz significantly reduced NO release by macrophages stimulated with LPS in a concentration that did not affect cell viability. This result corroborates a previous report that an extract from the leaves of *Alpinia zerumbet* reduced NO release in hepatocytes stimulated with interleukin 1 beta (IL-1β) [28]. EOAz also reduced NO release, but at a concentration that drastically reduced cell viability, which impairs drawing any conclusion. Interestingly, the Fb-Vehicle also reduced NO release in a non-cytotoxic concentration, which may be related to the antioxidant properties of some compounds present in its composition, such as vitamin E. However, the Fb-Vehicle did not show anti-hyperalgesic effects *in vivo*, suggesting a lack of correspondence with the *in vitro* assay, possibly related to the concentration used or the incubation time. Macrophages stimulated with LPS release several pro-inflammatory cytokines via the TLR4/NF-κB pathway, including IL-6. IL-6 injection induces heat and mechanical hyperalgesia [29] and its levels are altered in many painful conditions, including postoperative pain, rheumatoid arthritis, and temporomandibular joint disorders, among several others [30]. Fb-EOAz caused a concentration-dependent reduction of IL-6 levels in macrophages stimulated with LPS, reinforcing the idea of a potential anti-inflammatory effect. IL-6 signaling is downstream of Tumor necrosis factor-alpha (TNF-α) and requires the activation of the NF-κB pathway. Thus, further experiments are necessary to elucidate the specific target or targets of Fb-EOAz in the pro-inflammatory signaling cascade. In this regard, a previous study that assessed the effects of an extract from the fruits of *Alpinia zerumbet* showed that it disrupts the TLR4/NF-κB pathway in endothelial cells stimulated with LPS, reducing the expression of adhesion molecules and pro-inflammatory cytokines, including IL-6 and TNF-α [31]. It remains to be determined whether preparations using the leaves have a similar mechanism and to identify the specific targets of Fb-EOAz.

Finally, the effect of the different treatments was assessed in the open field test. The locomotory behavior of the rats, evaluated by the distance traveled and the average speed, was not influenced by the surgical procedure (sham or incision) or by any of the treatments, indicating that EOAz and Fb-EOAz do not cause hyperlocomotion or have a potential sedative effect. Likewise, the time spent by the animals in the center of the arena was not different between sham and incision rats, although the latter presented a high intergroup variation. A previous study from our group demonstrated that the rats subjected to the intraoral incision develop anxiety-like behavior on day 3 after the surgical procedure [13]. However, different from the present study, the anxiety-like behavior in mentioned study was assessed in the elevated plus maze (EPM) [13], which is considered more sensitive than the OFT for this analysis [17]. Interestingly, compared to the inert oil (*i.e.*, avocado oil), EOAz caused a significant reduction in the time spent in the center of the arena, which suggests a potential anxiogenic effect. This finding contrasts previous observations of an anxiolytic effect of the extract from the leaves of *Alpinia zerumbet*, but the doses, via an interval of administration, are different in the two studies, not allowing a direct comparison [32]. As already mentioned, the present result needs to be further explored by using complementary tests, such as the EPM. Both tests, OFT and EPM, are based on the natural aversion of rodents to exposed environments, but the EPM seems to create a more anxiogenic environment than the OFT, which explains its high sensitivity to detect anxiety-like behavior. Despite that, this result indicates another advantage of the Fb-EOAz compared to EOAz, since the commercial formulation did not change any of the parameters evaluated in the OFT.

One clear advantage of the present study is the route of administration of the compounds, which mimics clinical use, is non-invasive, convenient, safe, and easy to use, with less potential to cause systemic adverse effects and drug interaction. Thus, this treatment can bring benefits in orofacial pain control of inflammatory origin, with the advantage of a better safety profile compared to systemic treatments. However, one limitation associated with this treatment is the lack of pharmacokinetics studies, which would greatly contribute to the understanding of the mode of action of the compounds. Other potential limitations of the present study are the use of a single dose or concentration in the experiments and the lack of females’ inclusion. Regarding this later topic, it is noteworthy that our group had already characterized the incision model in male and female rats, and no sex differences were found in the time course or in the magnitude of evoked responses to heat and mechanical stimulation [15]. Nonetheless, the treatments’ efficacy on female rats needs to be determined.

## 5. Conclusions

In conclusion, the present study presented original data regarding antinociceptive and anti-inflammatory effects of the essential oil of *Alpinia zerumbet* compared to a ready-to-use commercial formulation based on the essential oil. The advantages found of the commercial formulation were the anti-hyperalgesic effects against mechanical hyperalgesia in the postoperative pain model and against heat hyperalgesia induced by LPS anti-inflammatory effects *in vitro* in a concentration that does not interfere with cell viability. Altogether, the results provide the first preclinical evidence of the efficacy of a formulation based on the essential oil of *Alpinia zerumbet* for the treatment of inflammatory orofacial pain conditions. In the clinical setting, this approach may represent an alternative or adjuvant therapy in the control of inflammatory and myofascial orofacial pain, which may be of great value, especially for patients for whom current systemic therapy is not recommended. Further studies are necessary to explore its mechanism of action, efficacy in other pain conditions, and safety.
